# Sex-specific differences of advanced glycation end products in diabetes

**DOI:** 10.1038/s41387-025-00379-6

**Published:** 2025-06-14

**Authors:** Michael Hellwig, Julia Decker, Leticia Prates Roma, Stefan Schunk, Emmanuel Ampofo, Sandra Rother

**Affiliations:** 1 Professur für Spezielle Lebensmittelchemie, Technische Universität Dresden, Dresden, Germany; 2 Institute of Biophysics, Center of Integrative Physiology and Molecular Medicine (CIPMM), Saarland University, Homburg, Germany; 3 Center for Gender-specific Biology and Medicine (CGBM), Saarland University, Homburg, Germany; 4 Department of Internal Medicine IV, Nephrology and Hypertension, Saarland University, Homburg, Germany; 5 Department of Nephrology, Medical Clinic 3, Klinikum Bamberg, Bamberg, Germany; 6 Institute of Clinical and Experimental Surgery, Saarland University, Homburg, Germany

**Keywords:** Risk factors, Cell biology

## Abstract

Advanced glycation end products (AGEs) are formed through non-enzymatic glycation reactions and accumulate in tissues, particularly under pathological conditions such as diabetes mellitus. These compounds are linked to the progression of diabetic complications, including nephropathy, retinopathy, and cardiovascular disease, through mechanisms such as oxidative stress and chronic inflammation. Emerging evidence suggests significant sex-specific differences in AGE formation, accumulation, and their biological effects, influenced by hormonal variations, dietary patterns, and metabolic differences. While the underlying biochemistry of AGE formation, such as the Maillard reaction and dicarbonyl compound activity, is well-characterized, the implications of these processes for clinical outcomes remain underexplored. This mini-review highlights the interplay between molecular mechanisms and sex-specific factors in AGE-related pathophysiology. It further discusses potential therapeutic approaches targeting AGE formation and receptor-mediated pathways, emphasizing the importance of integrating sex-specific considerations into diabetes management. Bridging molecular insights with clinical practice could advance personalized treatment strategies for diabetic complications.

## Introduction and brief overview of advanced glycation end products (AGEs) and sex-specific considerations

This mini-review provides an overview of the current understanding of sex-specific differences of individual AGEs in diabetes, encompassing their formation, mechanisms of action, and implications in disease progression. While this review centers on the role of AGEs in diabetes, where their formation is markedly accelerated under hyperglycemic conditions, it is important to note that AGEs are also critically involved in the pathophysiology of other chronic conditions, including cardiovascular diseases, neurodegenerative disorders, and chronic kidney disease [1–3]. Especially, sex-specific differences play a crucial role in fostering personalized medicine due to the inherent biological disparities between males and females. These distinctions extend to various physiological, genetic, and hormonal factors. Recognizing and understanding these differences is imperative for tailoring medical interventions to individual patient needs.

## Formation of AGEs and sex-specific differences in AGE levels

**Fig. 1:**
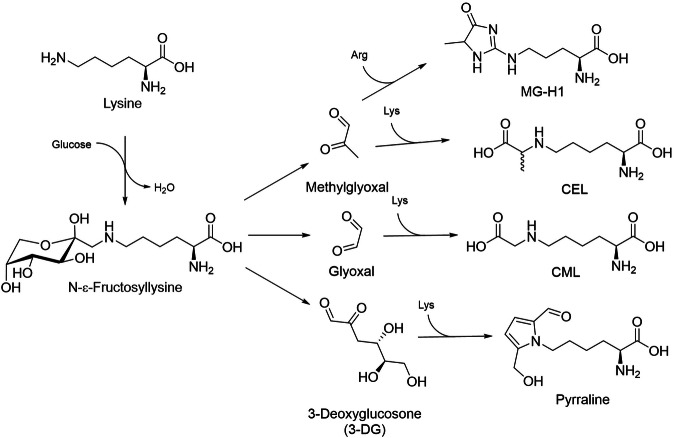
Scheme of the Maillard reaction (glycation). Arg arginine, CEL N-ε-carboxyethyllysine, CML N-ε-carboxymethyllysine, Lys lysine, MG-H1 methylglyoxal-derived hydroimidazolone 1.

The Maillard reaction (glycation) is a chemical process that starts with the reaction of a reducing sugar with an amino group of free amino acids, peptides and proteins (Fig. 1). The reaction is well known in food chemistry because it ultimately leads to the formation of different aroma compounds as well as the melanoidins that are responsible for the color of heated foods. Owing to this color formation, the reaction is also called “non-enzymatic browning”.

The first stable compounds formed in the reaction are called “Amadori rearrangement products (ARPs)” and retain a sugar amino-acid structure. These compounds are unstable during further heating. They are degraded to reactive 1,2-dicarbonyl compounds, of which 3-deoxyglucosone (3-DG) is the quantitatively most relevant compound in food [4]. However, small-molecular compounds such as methylglyoxal (MGO) and glyoxal (GO) may account for higher reactivity in food systems. The dicarbonyl compounds react with the side chains mainly of lysine and arginine to stable products such as N-ε-carboxy(m)ethyllysine (CML, CEL), methylglyoxal-derived hydroimidazolone 1 (MG-H1), and pyrraline (Fig. 1), which introduce permanent covalent modifications in food proteins. These substances are sometimes called “advanced glycation end products (AGEs)”.

Reducing sugars can also react on proteins in vivo, and the compounds fructosyl-lysine, CML, CEL, and MG-H1 (Fig. 1) have been detected in body fluids and proteins [5]. Concentrations of glycation compounds in the body tend to be higher in diabetes and uremia. Glycation of blood constituents by glucose as the main reducing sugar leads to two reaction products that are used diagnostically for the estimation of short- and long-term blood glucose concentrations. One is HbA_1C_, an Amadori rearrangement product formed at the N-terminal valine residue of the β-chain of hemoglobin. Due to hemoglobin having a half-life of approximately 120 days, HbA_1C_ is an integral marker for the mean blood glucose concentration in the preceding months. The other, fructosamine, represents Amadori rearrangement products formed at different sites of serum proteins and is a measure of the mean glucose concentration during the preceding weeks [6]. Even the formation of fluorescent compounds in the later stages of glycation may be applied diagnostically: skin autofluorescence as a measure of glycation reactions in tissues is a predictor of diabetic complications [7, 8].

Both endogenously produced and food-derived AGEs contribute to the overall AGE exposure, though their relative impact depends on factors such as concentration, metabolism, and clearance, since Maillard reaction products are a quite diverse set of structures. Compounds with a potential impact on human physiology are, e.g., dicarbonyl compounds such as GO, MGO, and 3-DG [9, 10].

### Sources of AGEs in diet and metabolism

AGEs can originate from both exogenous (dietary) and endogenous (metabolic) sources. Endogenous AGEs are produced continuously in the body through the Maillard reaction, but their accumulation increases with age and under conditions of hyperglycemia and oxidative stress [11]. Overall, both dietary and metabolic sources contribute to AGE accumulation in the body with the difference that dietary AGEs occur in a free form in the circulation, whereas endogenous AGEs mainly form on body proteins.

**Fig. 2:**
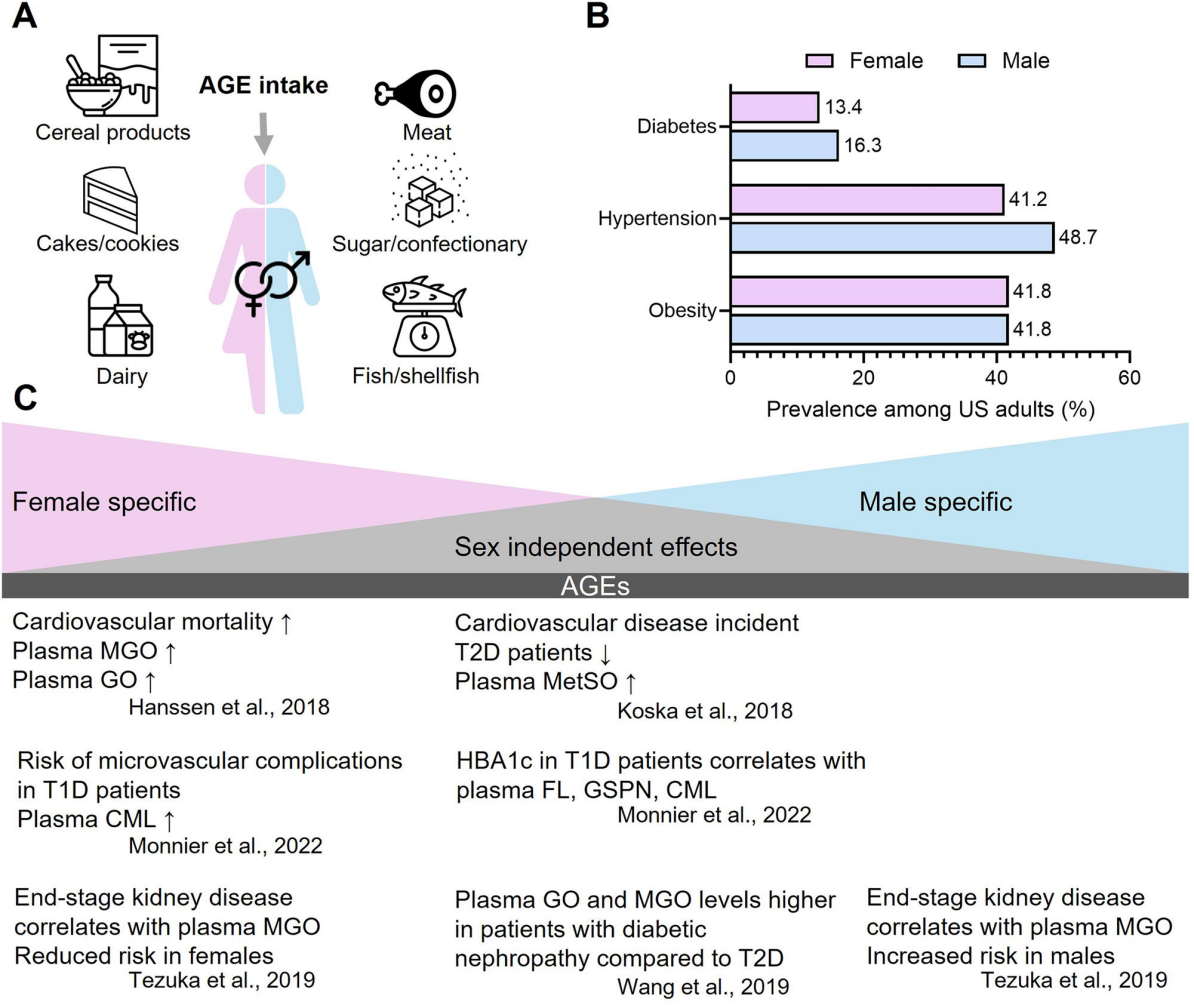
AGE intake and sex-specific differences of AGEs in diabetes and co-morbidities. **A** Main food groups responsible for the dietary uptake of AGEs. **B** Sex-specific differences in the prevalence of diabetes, hypertension and obesity among adults in the US [79]. **C** Selected sex-dependent and independent effects of AGEs in the context of diabetes.

Foods cooked at high temperatures, especially through dry heat methods like grilling, frying, roasting, and broiling, can lead to significant AGE formation. Thus, processed foods, including baked goods, snacks, and convenience foods, often contain high levels of AGEs. Moreover, cooked and processed animal-derived foods, such as meat, poultry, and high-fat dairy products, naturally contain AGEs. These foods typically have higher levels of AGEs such as CML, CEL and MG-H1 when compared to plant-based foods [12]. Overall, cereal products, meat, cakes/cookies, sugar and confectionery, dairy and fish/shellfish are the top six food groups responsible for the main AGE intake in Europe (Fig. 2A) [13].

Comparing the daily AGE intake (Table 1) based on an AGE database [12], no significant sex-specific differences were observed for diets following the USDA dietary guidelines [14]. However, several gender-specific preferences and dietary patterns were reported previously. Lombardo et al. [15] found that women consume more vegetable drinks, legumes, cooked vegetables, raw vegetables, cereals, whole grains and tofu. Men consume more meat, red meat, processed meat and eggs. Another study demonstrated sex differences in the mediterranean diet and reported that women have a higher carbohydrate, fruit and vegetable intake than men, who consume more animal proteins in the form of meat and eggs [16]. Furthermore, Feraco et al. [17] revealed that women consume more vegetables, whole gain, tofu, dark chocolate, fruit, non-dairy alternatives, while men eat more red and processed meat and eggs. When focusing on the younger population between 20 and 26 of age in Poland, women are known to consume more fruit, vegetables, fiber, whole meal bread, low-fat milk drinks and cottage cheese. Young men, on the other hand, eat a high-fat diet with more canned and meat dishes, fast food, cheese, potatoes and sugar in hot drinks [18]. In line with this, female university students in Germany reported to eat more cooked and raw vegetables, salad, fruit, fresh cheese, yoghurt and chocolate, whereas men consume more red meat, poultry, sausages, fish, hard and soft cheeses, fast food, pasta, rice, fried potatoes, and chips [19]. In summary, our calculations of the AGE intake based on the AGE database of Scheijen et al. [12] following reported gender-specific preferences and dietary patterns showed significant differences in the CML intake according to Lombardo et al. [15] and for the CEL intake between women and men considering the dietary differences described in the literature [15, 17–19]. However, no significant differences were observed for the other analyzed model diets. Since only three selected AGEs were considered, more potential sex-specific differences in the AGE intake according to dietary differences might exist.

**Table 1:** Differences in CML, CEL and MG-H1 uptake between men and women.

		Women	Men
	Reference	Mean ± SD	Mean ± SD
CML (mg/day)	USDA	2.61 ± 0.73	3.31 ± 0.98
	Lombardo et al. *(*p* = 0.019)	2.77 ± 0.97	4.35 ± 1.04
	Barrea et al.	2.75 ± 0.76	3.78 ± 0.95
	Feraco et al.	3.40 ± 0.89	4.27 ± 1.21
	Gil et al.	2.57 ± 0.79	3.33 ± 1.14
	Hilger et al.	3.16 ± 0.89	4.04 ± 1.14
CEL (mg/day)	USDA	1.65 ± 0.39	2.08 ± 0.35
	Lombardo et al. ***(*p* < 0.0001)	1.87 ± 0.51	3.53 ± 1.34
	Barrea et al.	1.73 ± 0.52	2.43 ± 0.36
	Feraco et al. *(*p* = 0.014)	1.83 ± 0.38	2.83 ± 0.73
	Gil et al. *(*p* = 0.030)	1.56 ± 0.37	2.47 ± 0.56
	Hilger et al. *(*p* = 0.033)	1.79 ± 0.39	2.69 ± 0.38
MG-H1 (mg/day)	USDA	20.51 ± 4.35	26.86 ± 6.00
	Lombardo et al.	26.88 ± 4.22	24.08 ± 5.08
	Barrea et al.	24.22 ± 5.25	24.26 ± 3.88
	Feraco et al.	24.28 ± 2.91	24.57 ± 4.01
	Gil et al.	21.75 ± 4.45	22.88 ± 4.12
	Hilger et al.	22.81 ± 4.86	25.29 ± 4.73

The daily intake was calculated based on the AGE database of Scheijen et al. [12]. The USDA dietary guidelines served as reference to calculate the AGE intake for 7 sample days assuming a calorie intake of 1800 kcal for women and 2400 kcal for men [14]. Additionally, the AGE intake was calculated following reported gender-specific preferences and dietary patterns [15–19]. Two-way ANOVA with Bonferroni´s multiple comparisons test.

*p < 0.05.

### Lifestyle and behavioral variations impacting AGE levels

While AGE formation occurs naturally in the body as part of normal metabolism, certain lifestyle and behavioral factors such as dietary habits, smoking, physical activity, alcohol consumption, obesity, stress and sleep patterns can accelerate glycation of susceptible endogenous proteins. Poor sleep quality in patients with type 2 diabetes mellitus (T2DM) was associated with enhanced HbA_1c_ levels [20]. Consumption of high-AGE foods, particularly those processed at high temperatures (such as fried, grilled, or roasted foods), can significantly elevate circulating levels of free AGEs. Additionally, diets rich in sugars can promote AGE formation through glycation reactions. Conversely, diets high in antioxidants may help mitigate AGE accumulation [21]. In a randomized clinical trial, elevated HbA_1C_ levels were significantly reduced by a low-carbohydrate diet in patients without taking glucose-lowering medication [22]. However, 75% of the participants were females, and no comparison between men and women was reported.

Excessive alcohol intake can contribute to AGE formation through several mechanisms, including reactive metabolites generated by cytochrome P450-2E1 (CYP2E1), leading to acetaldehyde adducts [23]. Chronic alcohol consumption is also associated with insulin resistance and altered glucose metabolism, further exacerbating AGE accumulation [11]. Regular exercise has been shown to reduce AGE levels by enhancing insulin sensitivity, promoting glucose uptake, and improving mitochondrial function. Conversely, sedentary behavior and physical inactivity are associated with higher circulating AGE levels and increased oxidative stress [21].

Obesity is characterized by chronic low-grade inflammation and oxidative stress, which can promote AGE formation and accumulation. Adipose tissue-derived cytokines such as tumor necrosis factor-α (TNF-α) and adipokines play a role in AGE metabolism and clearance, and their dysregulation in obesity can contribute to elevated AGE levels [11, 24, 25].

## Sex-specific role of AGEs in diabetes mellitus pathogenesis and co-morbidities

Persistent hyperglycemia in diabetes accelerates the production of AGEs, concomitant with the development of insulin resistance and diabetes complications such as cardiovascular disease, retinopathy and nephropathy including chronic kidney disease [26] (Fig. 2B). Thus, endogenously formed AGEs are discussed to contribute significantly to the development and progression of diabetes by disrupting cellular homeostasis and promoting a pro-inflammatory milieu. Once formed, AGEs can impact cellular function by modifying protein structure. Moreover, they are often described as interacting with both specific and non-specific AGE cell receptors. Among these, the most extensively studied receptor is the so-called “Receptor for Advanced Glycation End-products” (RAGE).

RAGE-mediated signaling regulates the progression of diabetic complications by driving chronic inflammation and tissue remodeling. RAGE, a multiligand receptor of the immunoglobulin superfamily, is activated by AGEs and other damage-associated ligands. Binding to RAGE triggers intracellular signaling cascades, notably activating nuclear factor kappa B (NF-κB), which promotes the transcription of pro-inflammatory cytokines and further upregulates RAGE itself. This feed-forward loop sustains inflammation, contributing to cell migration, proliferation, fibrosis, and extensive tissue damage. In diabetes, hyperglycemia increases AGE accumulation in renal tissues, amplifying RAGE signaling [27]. While RAGE-ligand interactions are central to this process, the precise role of glycation remains incompletely understood. Glycation-induced oligomerization of proteins seems more relevant for RAGE binding than the formation of individual glycated amino acids [28, 29].

Following, we will summarize how AGEs impact different signaling pathways with a focus on diabetes and how biological sex affects this scenario (Fig. 2C).

Experimental evidence from in vitro and in vivo studies highlights the detrimental effects of glycated proteins on pancreatic beta cells, insulin signaling, and vascular function, exacerbating hyperglycemia and insulin resistance. For example, treatment of INS-1 cells with glycated bovine serum albumin enhanced cell apoptosis and reactive oxygen species (ROS) production [30].

In diabetes, sex-specific factors play a crucial role in disease manifestation and progression [31, 32]. While men have a higher absolute rate of cardiovascular complications, women with diabetes experience a greater relative increase in risk compared to men. Studies have shown that diabetes and prediabetes disproportionately amplify cardiovascular risk in women, reducing their usual protective advantage and leading to a higher relative risk of myocardial infarction and heart failure [33, 34].

Overall, menopause is linked to increased central adiposity and insulin resistance, raising T2DM risk. Diabetes may also accelerate ovarian aging, with earlier menopause further increasing T2DM risk. Hormone therapy can help reduce this risk and improve glycemic control [35]. Many menopause-related metabolic risks can be mitigated through dietary modification [36]. While AGEs play a key role in diabetes pathophysiology and are diet-sensitive, their role in menopause remains underexplored. Understanding these links may clarify sex-specific AGE-related mechanisms in midlife metabolic health.

Hormonal variations, including fluctuations in estrogen levels, can impact insulin sensitivity and glucose metabolism differently in females compared to males. AGE levels are increased in women after menopause, and this effect is magnified in diabetic post-menopausal women [37, 38]. However, the latter data were obtained by immunochemical measurement of AGEs, which, while widely used and valuable, may lack specificity in distinguishing individual AGE compounds in complex biological samples. Given the known variability in ELISA-based detection, assay calibration and epitope specificity, complementary validation using chromatography-based methodologies, such as liquid chromatography-mass spectrometry (LC-MS), can enhance accuracy and specificity [39, 40]. Therefore, further confirmation of these findings with chromatography-based approaches would strengthen their reliability.

Plasma CML concentrations in patients with type 1 diabetes (T1DM) were significantly associated with sex, while no sex-specific association was found for fructosyl-lysine, glucosepane, MG-H1, CEL, G-H1, pentosidine, MOLD, 3-nitrotyrosine, o-tyrosine and plasma methionine sulfoxide (MetSO) [41, 42]. On the other hand, higher plasma MetSO concentrations in patients with T2DM were associated with a lower risk of incident cardiovascular disease events independent of sex [42].

### Oxidative stress

Sex-specific differences in oxidative stress are influenced by a complex interplay of hormonal, genetic, and physiological factors, resulting in distinct susceptibilities to oxidative damage between males and females. Oxidative stress, defined as an imbalance between the production of ROS and antioxidant defenses, is a key contributor to cellular damage and is implicated in aging and numerous pathologies, including diabetes. On the other hand, ROS also act as signaling molecules, involved in physiological responses to intra- or extracellular cues.

RAGE has been identified as a critical factor of sex-specific differences in murine kidney injury, with male RAGE knockout mice exhibiting greater renal damage and fibrosis than females, potentially due to the lack of estrogen-mediated protection [43]. In addition, RAGE deficiency induced sex-specific insulin resistance, accompanied by upregulation of antioxidant and browning-associated genes in female adipose tissue [44]. Of note, plasma soluble RAGE was recently described as a predictor of insulin sensitivity, suggesting a possible function in glycemic control maintenance. However, no sex-specific differences were reported [45].

**Fig. 3:**
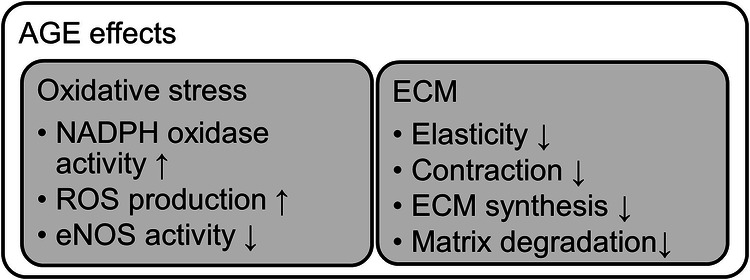
Overview of selected biological effects of protein glycation.

Several studies have already shown that RAGE activation can stimulate the nicotinamide adenine dinucleotide phosphate (NADP)H oxidase enzyme complex, which is responsible for the NADPH-dependent reduction of molecular oxygen (O₂) into the superoxide anion (O₂^•−^), a reactive form of oxygen [46–48] (Fig. 3). In addition, mitochondrial ROS (O₂^•−^ and H_2_O_2_) have also been reported upon RAGE activation, leading to changes in cellular respiration and proton leak. Interestingly, O₂^•−^ production via NADPH oxidases has been shown to be more pronounced in the endothelium of male versus female rats under basal conditions [49]. In addition, in mice fed with a high-fat diet, RAGE deficiency prevents oxidative stress in adipose tissues of females but not in male, which was in parallel with an increase in antioxidant enzymes (catalase, superoxide dismutase 2, and glutathione peroxidase 1) [44].

Previously, we have shown that short-term (24-48 h), AGEs (albumin modified by glycolaldehyde) lead to protection against apoptosis in cultured pancreatic islets compared to control islets cultured with non-modified albumin. However, longer exposure time (72-96 h) leads to an increase in oxidative stress due to NADPH oxidase activation and increased apoptosis. NADPH oxidase and O₂^•−^ production precedes apoptosis and might be involved in the shift between protective versus deleterious effects [48].

AGEs are implicated in the formation of reactive nitrogen species (RNS) through increased NO production from inducible nitric oxide synthase (iNOS) [50]. In contrast, Lauer et al. found no effects of free or albumin-bound AGEs on NO availability. Moreover, they reported that protein-bound AGEs can decrease nitrite concentrations [51]. The reaction between NO and superoxide (from NADPH oxidases) forms peroxynitrite (ONOO⁻), a potent oxidizing and nitrating agent that modifies proteins and disrupts cellular function. Peroxynitrite can also uncouple endothelial NOS (eNOS), shifting its activity from NO production to further superoxide generation, thereby intensifying oxidative and nitrosative stress [52]. The decline in eNOS activity and subsequent endothelial dysfunction are more pronounced in post-menopausal women due to estrogen loss [53]. By using immunochemical methods, higher CEL levels and oxidative stress were reported for male rats compared to female rats, which might contribute to the sex-specific differences in hypertension development [54]. This correlation will also need independent confirmation by chromatography-based AGE quantification.

### Extracellular matrix effects

The extracellular matrix (ECM) is a 3-dimensional scaffold synthesized by the local cells of a tissue composed of structural proteins such as collagen, fibronectin, glycoproteins, and proteoglycans, providing essential biochemical and physical cues for tissue homeostasis [55]. In diabetes mellitus, MGO modification represents the major protein modification by glycation of both intra- and extracellular proteins [5]. ECM glycation was shown to contribute to diabetic microvascular complications (Fig. 3). AGE-modified ECM proteins can form inter- and intramolecular crosslinks within structural proteins like collagen and elastin, fostering arterial and dermal stiffness and altering endothelial cell behavior [56]. Overall, ECM protein glycation can decrease the vascular elasticity, enhancing vascular inflammation and permeability and fostering pericyte apoptosis [11, 57]. Hanssen et al. [58] measured plasma AGE levels in patients with T2DM and found a correlation between MGO and cardiovascular disease incidence. Interestingly, they stratified the hazard ratio analysis according to sex. The authors found that MGO and GO plasma levels were significantly more strongly associated with cardiovascular disease mortality and total mortality in women when compared to men [58]. However, there were no sex-specific associations between MGO, GO and 3-DG levels and cardiovascular disease incidence.

AGEs have been shown to affect the expression of ECM proteins and matrix-degrading matrix metalloproteinases [59]. Of note, collagen glycation increases the resistance against enzymatic degradation, resulting in a further increase of AGEs in the ECM [60]. Since collagen, as the main structural protein of the skin ECM, possesses a high longevity, its glycation increases over time with age. In a cross-sectional study measuring the skin autofluorescence as a readout for AGEs, Birukov et al. [61] found that the association between skin autofluorescence and vascular stiffness occurred only in men and was consistent across different glycemic states. However, no detailed AGE profiling in the skin or in the plasma was performed [62]. Mook-Kanamori et al. [63] stressed that sex differences should be considered for AGEs measured by skin autofluorescence since they observed significantly higher values in females. In contrast, another study observed higher skin fluorescence values in men than women [64]. These authors suggested that estrogen might be a protective factor against AGE formation and accumulation even later in life. However, other factors like differences in skin color as well as lifestyles (e.g., smoking) can further influence those measurements. CML was also found in the bone ECM. However, due to the small group of 5 donors (2 female, 3 male) with wide age differences, no assessment of gender differences was possible [65].

Elevated AGEs and alterations in ECM composition, including changes in glycosaminoglycans (GAGs), play a significant role in the pathophysiology of diabetes and its complications [66, 67]. Besides ECM proteins, GAGs, as a functional part of proteoglycans, are the main constituents of the ECM, directing cell-cell and cell-matrix interactions [68]. Elevated AGEs are reported to be associated with plasma GAGs. While no significant differences in the plasma GAG levels of chondroitin sulfate and dermatan sulfate between males and females were observed, plasma heparan sulfate concentrations correlated with age in women [69].

## Clinical implications and therapeutic strategies

Lifestyle interventions like adopting healthy cooking methods, maintaining blood glucose levels within a healthy range, reducing oxidative stress and avoidance of smoking are the standard therapy in an early stage of T2DM [10, 70]. However, when this therapeutic strategy is not sufficient to lower blood glucose levels to a physiological range, patients with T2DM are treated with various anti-diabetic drugs such as biguanides that lower blood glucose levels by inhibiting hepatic gluconeogenesis and glucagon reduction [71]. Interestingly, targeted intervention strategies decreasing AGE accumulation, blocking AGE formation, as well as RAGE-dependent inflammation reactions, are currently explored for the treatment of T2DM [72]. For instance, aminoguanidine inhibits AGE formation and was shown to reduce T2DM-associated cardiac fibrosis in rats [73]. Furthermore, Thornalley [74] detected AGEs in endoneurial microvessels as well as myelinated and unmyelinated fibers of human diabetic subjects. Accordingly, the use of RAGE antagonists may be a suitable strategy to reduce diabetic neuropathies. Interestingly, the reduction of RAGE-mediated signaling not only has a positive impact on the progression of T2DM but may also be able to improve the outcome of islet transplantation, which is a therapeutic option for patients with T1DM with severe hypoglycemia or after kidney transplantation [75]. However, many transplanted islets die due to ischemia and immunosuppression in the early post-transplant phase [75]. In this context, the group of Herold demonstrated the crucial role of RAGE in the activation of adaptive immune responses to auto- and alloantigens [76]. They could show that the specific inhibition of RAGE markedly delays allograft rejection. These results were confirmed by the study of Matsuoka [77], reporting that RAGE is involved in the early loss of transplanted islets mediated by the binding of high-mobility group box 1 (HMGB1) to this receptor. Moreover, Zhang et al. [78] deciphered the underlying molecular mechanism by demonstrating that HMGB1 activates RAGE, resulting in an enhanced phosphatidylinositol 3-kinase (PI3K)-Akt-mechanistic target of rapamycin (mTOR) signaling and, thus, impaired Treg stability and functionality.

All these preclinical studies revealed that endogenously formed AGEs play a crucial role in the development and progression of diabetes. However, future clinical studies are required to show the potential of lowering AGE-mediated signaling for the treatment of patients with T2DM and T1DM.

## Outlook and conclusion

Overall, AGE-mediated modifications of proteins within the body can impair tissue integrity and function, exacerbating diabetic nephropathy, retinopathy, neuropathy, and cardiovascular complications. Understanding the intricate interplay between AGEs and diabetic complications may unveil novel therapeutic strategies for mitigating disease burden. Even though it is often stressed that AGE profiling of specific AGEs is required for detailed structure-function analysis, the prerequisite of special equipment and expertise has been a limiting factor in the past. Increasing evidence points to the potential use of specific AGEs as biomarkers that might predict diabetic complications. However, further studies on sex-specific differences of AGE-driven effects using up-to-date methodology for the quantitation of AGEs are required.
